# SDS-PAGE-Based Quantitative Assay of Hemolymph Proteins in Honeybees: Progress and Prospects for Field Application

**DOI:** 10.3390/ijms241210216

**Published:** 2023-06-16

**Authors:** Gloria Isani, Elisa Bellei, Cecilia Rudelli, Riccardo Cabbri, Enea Ferlizza, Giulia Andreani

**Affiliations:** 1 Department of Veterinary Medical Sciences, Alma Mater Studiorum-University of Bologna, Via Tolara di Sopra 50, Ozzano dell’Emilia, 40064 Bologna, Italy; gloria.isani@unibo.it (G.I.); cecilia.rudelli2@unibo.it (C.R.); rccabbri@gmail.com (R.C.);; 2 Department of Surgery, Medicine, Dentistry and Morphological Sciences with Transplant Surgery, Oncology and Regenerative Medicine Relevance, Proteomic Lab, University of Modena and Reggio Emilia, 41124 Modena, Italy; elisa.bellei@unimore.it; 3 Department of Medical and Surgical Sciences, Alma Mater Studiorum-University of Bologna, Via Belmeloro, 8, 40126 Bologna, Italy

**Keywords:** proteomics, nutritional biomarkers, vitellogenin, apolipophorin, hexamerin 70a, transferrin

## Abstract

In human and veterinary medicine, serum proteins are considered to be useful biomarkers for assessing the health and nutritional status of the organism. Honeybee hemolymph has a unique proteome that could represent a source of valuable biomarkers. Therefore, the aims of this study were to separate and identify the most abundant proteins in the hemolymph of worker honeybees to suggest a panel of these proteins that could represent useful biomarkers for assessing the nutritional and health status of the colonies and, finally, to analyze them in different periods of the year. Four apiaries were selected in the province of Bologna, and the bees were analyzed in April, May, July, and November. Thirty specimens from three hives of each apiary were sampled and their hemolymph was collected. The most represented bands obtained after 1D sodium-dodecyl-sulfate-polyacrylamide gel electrophoresis (SDS-PAGE) were cut from the gel, and the proteins were identified using an LC-ESI-Q-MS/MS System. A total of twelve proteins were unmistakably identified; the two most abundant proteins were apolipophorin and vitellogenin, which are known biomarkers of bee trophic and health status. The two other proteins identified were transferrin and hexamerin 70a, the first being involved in iron homeostasis and the second being a storage protein. Most of these proteins showed an increase from April to November, mirroring the physiological changes of honeybees during the productive season. The current study suggests a panel of biomarkers from honeybee hemolymph worth testing under different physiological and pathological field conditions.

## 1. Introduction

Insect hemolymph is composed of circulating cells and fluid containing proteins, lipids, carbohydrates, nucleic acids, hormones, and ions [1,2]. In human and veterinary medicine, serum proteins, either total proteins or specific proteins such as albumin, are considered important biomarkers for assessing the health and nutritional status of an organism.

Gel electrophoresis is a useful tool for separating and analyzing proteins, from agarose gel electrophoresis used to separate serum proteins in clinical routine to the more sensitive mono (1D)- or bidimensional (2D) sodium dodecyl sulfate–polyacrylamide gel electrophoresis (SDS-PAGE) used to separate complex protein mixtures of different biological samples [2,3,4,5]. The clinical applications of electrophoresis in separating hemolymph proteins are still limited in honeybees due to the paucity of data defining a reference proteome. In addition, there are two main issues complicating the interpretation of the results: the first is the difficulty of obtaining homogeneous samples from healthy animals, while the second is the high variability, accentuated by several factors such as polyethism or seasonal variations. Studies regarding hemolymph proteins have recently been reported; they focused on the differences between pupae and newly emerged bees using 2DE [2,6] or on *Nosema*–honeybee interaction [7]. However, due to the complexity of data, these studies do not offer the possibility of using their results in field applications. Moreover, despite its higher resolution power, 2DE cannot be routinely used due to its complex and time-consuming protocol. On the other hand, even if 1DE does not perform as well, its one-day protocol and its easier execution make it a valid and reliable method for studying hemolymph proteins and obtaining useful information on the health and nutritional status of honeybees.

Only a few diseases of the hive can be diagnosed by means of direct examination; for this reason, hive assessment is routinely coupled with laboratory analysis of hive matrix samples. On the other hand, there is a simple method for measuring the strength of a honeybee colony, the Liebefeld method which estimates the number of adult workers, brood, and food storage quantities [8]. However, methods for accurately assessing health and nutritional status at the colony level using molecular biomarkers are currently lacking. Therefore, the aims of this study were: (1) to separate and identify the most abundant and relevant hemolymph proteins of worker honeybees using an expeditious 1DE proteomic approach, (2) to suggest a panel from these hemolymph proteins which could represent a useful tool for measuring the nutritional and health status of the colony, and (3) to quantify these proteins in bees from different apiaries of the province of Bologna in different periods of the year. To overcome individual variations, a pooled sample of hemolymph from young worker honeybees was used, as suggested by Ricigliano et al. [9]. The use of a comprehensive panel of hemolymph biomarkers has never been proposed before and will represent meaningful advances in the preservation of bee populations.

## 2. Results and Discussion

### 2.1. The Total Protein Concentration in Hemolymph Varies Depending on the Season

**Figure 1:**
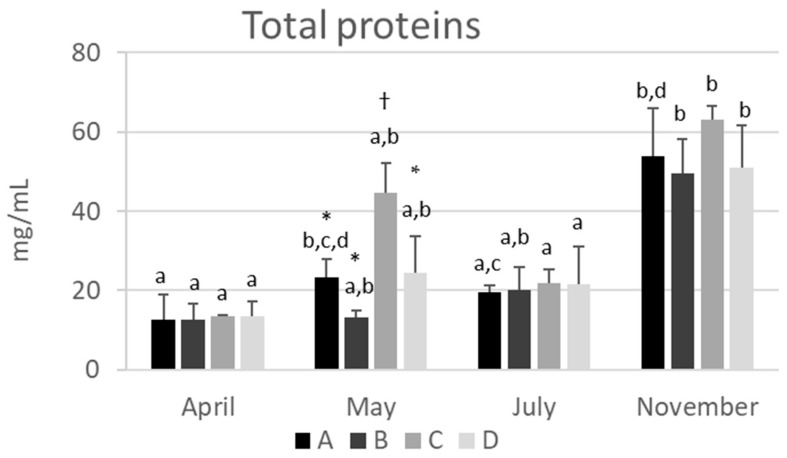
Seasonal variations of total protein concentrations in hemolymph of honeybees from four apiaries (A, B, C, and D) of the province of Bologna. The data are expressed in mg/mL and reported as the mean ± SD (n = 3). Different lower-case letters indicate significant differences (*p* < 0.05) among the time points (months) within the same apiary. Different symbols (*,†) indicate significant differences (*p* < 0.05) among the apiaries at the same time point (May).

The mean concentration of the total proteins in the hemolymph of the samples analyzed ranged from 13.0 ± 3.56 mg/mL in April to 54.4 ± 9.62 mg/mL in November. No significant differences were detected among the apiaries at the same sampling time, except for apiary C, which showed significantly higher concentrations of total proteins in May than all the other apiaries (*p* < 0.01) (Figure 1 and Supplementary Table S1). This finding can be related to the abundance of pollen in the surrounding semi-natural habitat rich in wild plants characterized by spring blooms, including acacia and linden trees. In all other months, the total protein values were homogeneous among the apiaries regardless of the environmental characteristics.

Hemolymph proteins showed a clear and significant (*p* < 0.05) increase from April to November in all the apiaries studied (Figure 1 and Supplementary Table S1), except for apiary C. The values obtained in the present study were consistent with those published by other authors. The mean values determined in July (from 19.6 ± 1.72 to 21.7 ± 3.60 mg/mL) are included in the range of those reported by Cabbri et al. [10] (from 12.4 ± 2.80 to 13.6 ± 2.90 mg/mL) in healthy colonies sampled in July 2017 in the province of Bologna and those around 30 mg/mL determined in summer in a colony located in an apiary near Brno, Czech Republic, by Kunc et al. [11]. These authors reported that the protein concentration increased significantly in the hemolymph starting from autumn and remained at over 50 mg/mL from October to March [11]. Accordingly, in the present study, the mean values determined in November varied from 51.1 ± 10.5 to 63.1 ± 3.49 mg/mL. In contrast, Dostálková et al. [12] found a concentration of hemolymph proteins of 34.5 in summer and 30.2 mg/mL in winter bees from the Czech Republic.

In previous papers, the total proteins in the hemolymph have been correlated with nutritional status [10,13,14]. A positive correlation with the quantity and quality of protein food intake was consistently found. The study of Basualdo et al. [15] showed that a diet based on bee bread promoted a significant increase in hemolymph proteins as compared to a protein substitute, and increased survival in bees artificially infected by *Nosema ceranae*. These results supported the hypothesis that the reaction of bees to pathogens was positively related to hemolymph proteins.

Future research about the use of total proteins as an index for assessing nutritional and health status is needed, as it could represent a simple and useful index, mirroring the quantification of serum total proteins in human and veterinary clinical biochemistry.

### 2.2. Identification of the Most Abundant Hemolymph Proteins Using Mass Spectrometry

**Figure 2:**
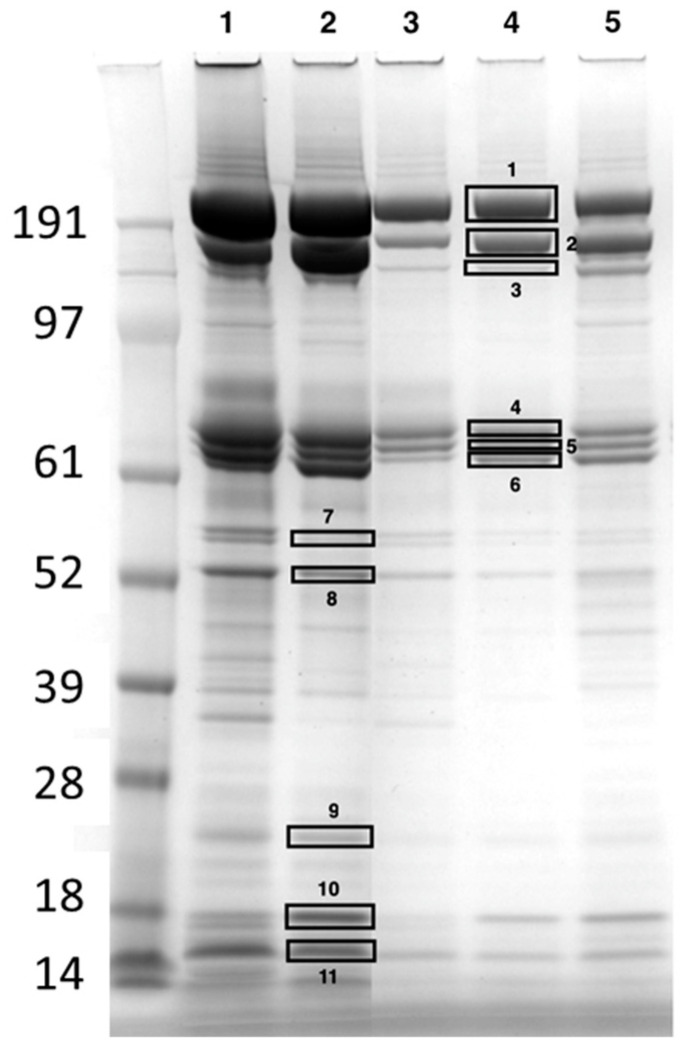
Representative gel obtained after SDS-PAGE (4–12%, Coomassie staining) of hemolymph proteins from healthy worker bees (apiary D, April sampling) at different dilutions (lanes 1–2, 15 µg; lanes 3–5, 3 µg). The first lane shows the molecular mass marker (kDa). The rectangles indicate the bands cut and analyzed using mass spectrometry that led to the protein identifications (Table 1). The numbers next to the rectangles correspond to those reported in Table 1.

To shed more light on the complexity of the hemolymph proteome, the proteins were separated using SDS-PAGE electrophoresis, and the most abundant bands were cut from the gel for subsequent identification using mass spectrometry. As an example, a gel showing the protein bands is reported in Figure 2 and Supplementary Figure S1. The bands had apparent molecular masses (MMs) ranging from >200 to 14 kDa. A total of 12 proteins were unmistakably identified (Table 1).

The two most abundant high MM bands contained apolipophorin (>200 kDa) and vitellogenin (180 kDa) (bands 1 and 2, respectively, Figure 2). Apolipophorin was also present at lower MMs in bands 4 (80 kDa) and 9 (21 kDa), and vitellogenin was also found in band 3 (150 kDa). In the middle part of the gel, other important proteins were identified: transferrin (70 kDa) and hexamerin 70a (67 kDa), present in bands 5 and 6, respectively. Prophenoloxidase (proPO) (58 kDa) and major royal jelly protein (MRJP) (54 kDa) were identified in bands 7 and 8, respectively. Finally, different isoforms of odorant binding proteins (OBPs), namely proteins which bind olfactory molecules, were identified in bands 9, 10, and 11. The other proteins identified are reported in Table 1.

Apolipophorins are multifunctional high MM proteins devoted to the transport of hydrophobic molecules and are integral components of the lipophorin systems, the major role of which is the transport of diacylglycerol, phospholipids, sterols, and hydrocarbons in the hemolymph. The honeybee lipophorins contain two integral apolipoproteins, apolipophorin I (240 kDa) and apolipophorin II (80 kDa), which derive from the same precursor apolipophorin I/II and may contain an exchangeable protein, apolipophorin III (18 kDa) [16]. Using the electrophoretic protocol developed in this study, it was possible to separate and identify apolipophorin I (band 1) and apolipophorin III (band 9) while the apolipophorin (fragment) present in band 4 (MM 80 kDa) should be apolipophorin II.

Vitellogenin is a complex 180 kDa monomeric phospholipoglycoprotein with a key trophic function; in fact, it has been shown to serve as a lipid transporter [17] and as the substrate from which nurse workers derive royal jelly [18]. It is released into the hemolymph from the fat body, the site of production and storage. From the hemolymph, vitellogenin passes to the acini of the hypopharyngeal glands which are devoted to the secretion of royal jelly [19]. The presence of a 150 kDa vitellogenin in band 3 seems to contradict what Havukainen et al. [20] hypothesized, namely that only the intact 180 kDa protein is present in worker hemolymph.

Transferrin is the main iron transport protein in all animals and is one of the most important proteins involved in maintaining homeostasis of this essential element. In insects, this monomeric protein of approximately 70 kDa transports iron from the gut to the fat body where this metal is stored in the trophocytes [21].

As in all holometabolous insects, the larva-to-pupa transition occurs at the expense of the protein reserves accumulated during the larval phase. These proteins are typically represented by hexamerins which occur in massive amounts in the hemolymph of larvae [22,23]. Genes encoding hexamerin are actively transcribed in the fat body of the larvae; the proteins are then released into the hemolymph where they represent an important source of amino acids during the pupal stage. Even in adult worker bees, hexamerin production continues in the fat body and may be involved in the transition between nurse and forager bees [24]. The hexamerin 70a detected in the hemolymph of the adult bees analyzed in this study is in agreement with the data reported by Martins et al. [24] and Danty et al. [25]. The absence of hexamerin 70b confirms the data of Chan et al. [22] who reported that the queen was the only caste showing hexamerin 70b during adulthood.

The other proteins identified were proPO, chitinase-like protein Idgf4, MRJP, and OBPs. The most abundant protein in band 7 was proPO, the inactive zymogen of phenoloxidase which controls the melanisation of pathogens and damaged tissues and represents one of the major innate defense systems in invertebrates. Activation of this enzyme is brought about by the proPO-activating system containing proteins capable of binding to polysaccharides and other compounds typically associated with microorganisms [26]. Chitinase-like protein Idgf4 belongs to a small glycoprotein family of chitinase-related secretory proteins found in various insect species; similar glycoproteins have been associated with antimicrobial responses and insect hemolymph clotting as well as with the molting process and extracellular matrix formation [27]. Major royal jelly proteins are the most abundant proteins in royal jelly. They can be present in monomeric form with an MM of approximately 55 kDa or join to form pentameric polymers. These proteins are secreted by the hypopharyngeal glands of the nurse bees and have important functions for insect development [28]. Finally, OBPs are small acidic proteins (~13–16 kDa) present in very high concentrations (10–20 mM) at the interface between the olfactory receptors and the external environment [29]. Their physiological role has not yet been elucidated; however, they have been associated with the transfer of olfactory molecules to membrane receptors [30].

### 2.3. Quantification of the Most Abundant Hemolymph Proteins

Hemolymph samples of bees obtained from four apiaries of the province of Bologna in April, May, July, and November 2022 underwent SDS-PAGE fractionation. The presence of two high-abundance proteins (apolipophorin and vitellogenin) represented an analytical problem, the same as albumin in mammal serum. As a matter of fact, loading a high protein concentration to 1DE while favoring the separation and quantification of less abundant proteins complicated the accurate quantification of vitellogenin and apolipophorin due to protein overload in these bands (Figure 2). Therefore, it was decided that a good compromise between high- and low-abundance proteins would be to load 3–5 µg of total proteins onto the gel.

**Figure 3:**
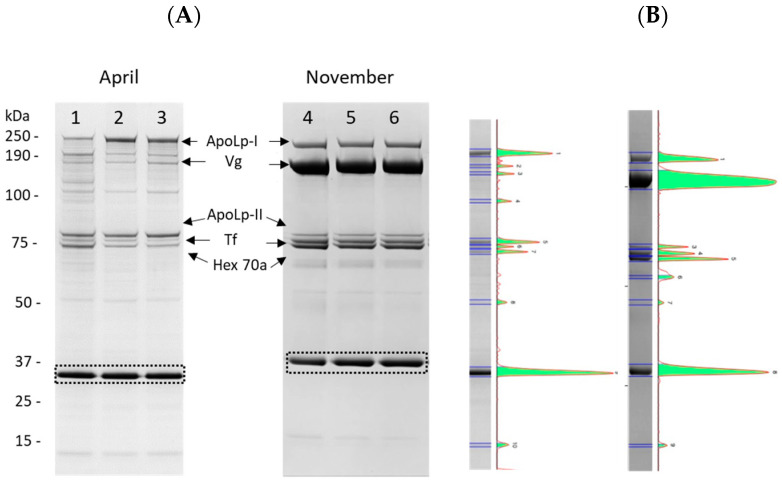
(**A**) Representative gels obtained after SDS-PAGE (4–12%, Coomassie staining) of hemolymph from worker bees sampled in April and November from the same 3 hives located in the province of Bologna (apiary A); the dashed black box indicates the internal standard of quantity (1 µg). (**B**) The pherograms of lanes 2 and 5 are reported as an example. The arrows indicate the bands identified as apolipophorin-I (ApoLp-I), vitellogenin (Vg), apolipophorin-II (ApoLp-II), transferrin (Tf), and hexamerin 70a (Hex 70a).

Apolipophorin-I and II, vitellogenin, transferrin, and hexamerin 70a could clearly be separated, detected, and quantified. As an example, gels and pherograms after SDS-PAGE separation of hemolymph samples obtained from the same colonies of apiary A in April and November are reported in Figure 3. The profiles obtained agreed with those reported after 1D SDS-PAGE by Chan et al. [22] and Cabbri et al. [10] for the hemolymph of worker honeybees, confirming the presence of a common set of the most abundant proteins. Complete original gel images are reported in Supplementary Figure S2.

**Figure 4:**
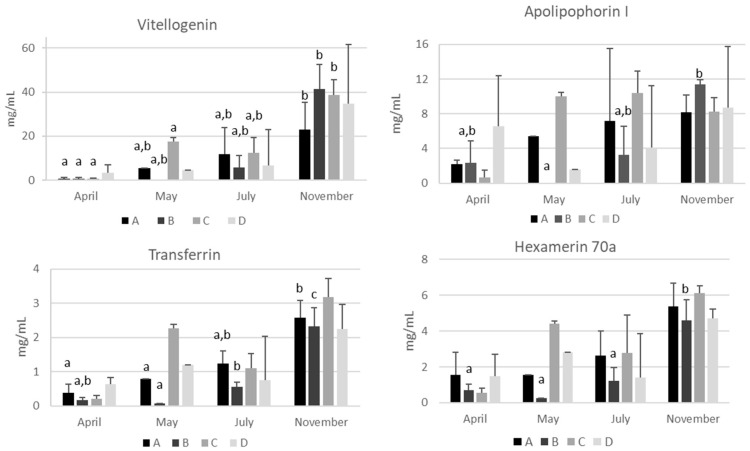
Seasonal variations of vitellogenin, apolipophorin-I, transferrin, and hexamerin 70a in the hemolymph of honeybees from four apiaries (A–D) of the province of Bologna. The data are expressed as mg/mL and reported as the mean ± SD (n = 3). For each analyte, different lower-case letters indicate significant differences (*p* < 0.05) among time points (months) within the same apiary.

Despite the variability among different colonies of the same apiary, quantitative differences are clearly visible across the seasons. Data regarding seasonal variations of apolipophorins, vitellogenin, transferrin, and hexamerin 70a are reported in Figure 4 and Supplementary Table S1. An increasing trend of these proteins from April to November is visible, mirroring the increase in hemolymph total proteins.

The mean concentrations of vitellogenin found in the present study varied from a minimum of 0.00 mg/mL in May in apiary B to a maximum of 41.5 ± 10.9 mg/mL in November in apiary B (Figure 4) and were consistent with those reported by Dostálková et al. [12] in summer and winter bees from the Czech Republic. A significant increase from April to November (*p* < 0.05) was detected in all the apiaries with the exception of apiary D (Figure 4 and Supplementary Table S1). Vitellogenin is the best known and the most studied hemolymph protein which has been proposed as a biomarker of bee trophic status and health [10,31]. Hemolymph vitellogenin is useful in establishing the vitality of the colony; it correlates with food quality [32], immune function [33], and/or exposure to pesticides [34]. A decrease in vitellogenin may also be related to loss of colony vitality [35]. Finally, increased vitellogenin concentration has been correlated with increased longevity [31]. It is known that vitellogenin is higher in winter bees when compared to summer bees [11,36]. Accordingly, the presence of a winter bee phenotype characterized by a high concentration of vitellogenin was also evidenced in this study in all the colonies analyzed, suggesting that this storage and antioxidant protein is essential to face the winter season, because it is probably used as a source of amino acids to sustain energy metabolism during the winter.

Regarding apolipophorins, significant variations were detected only in apiary B, with an increase from May to November (*p* < 0.05) (Figure 4 and Supplementary Table S1). These proteins transport lipids from the site of absorption (midgut) to the site of storage (fat body) and vice versa from the fat body to the tissues [37]. Their concentration in the hemolymph is closely related to lipid mobilization, and variations can be due to a number of causes, such as embryogenesis, immune response, and starvation [38]. A high apolipophorin concentration is a signal that should be carefully evaluated and not underestimated because lipid mobilization from the fat body is an index of malnutrition. Moreover, in honeybee colonies there is a strict correlation between nutritional status and immunity [39]. Therefore, apolipophorins can represent a good biomarker of lipid metabolism, monitoring at the same time the nutritional status and thus the honeybee resilience to pathogens.

Despite the importance of iron in honeybee physiology and metabolism [40], transferrin has been poorly investigated. Insect transferrin is considered a multifunctional protein because in addition to its involvement in iron metabolism other roles have been suggested [41]. Recently, Iatsenko et al. [42] reported that *Drosophila melanogaster* triggers a hypoferremic response after microbe infection. This response is aimed at limiting the availability of iron to pathogens and is part of the so-called nutritional immunity [43]. Accordingly, the study of Rodriguez-Garcìa et al. [40] evidenced that *Nosema ceranae* infection determined iron loss in infected bees suggesting altered iron homeostasis. In the present study, hemolymph transferrin increased significantly from spring to autumn (*p* < 0.05) (Figure 4 and Supplementary Table S1) in all the apiaries with the exception of apiary D with values ranging from 0.07 ± 0.01 mg/mL in apiary B in May to 3.18 ± 0.54 mg/mL in apiary C in November. The significance of this increase is currently unknown. It can be speculated that elevated transferrin concentrations represent increased iron-sequestrating capacity in case of microorganism infection limiting iron availability or, alternatively, an effective system for maintaining active iron trafficking between the fat body and the peripheral tissues during the cold season without the risk of oxidative stress. Therefore, the quantification of hemolymph transferrin could be of interest in assessing bee health status and deserves more research in the future.

Finally, despite an increase in hexamerin 70a being evident from April to November in all the apiaries analyzed, the difference was statistically significant only in apiary B (Figure 4 and Supplementary Table S1) due to high variability among the colonies of the same apiary. Danty et al. [25] observed that nutrition significantly influenced the accumulation of this protein in adult honeybees, at both the transcript and protein level. High concentrations of this protein in May in apiary C support a relationship with nutritional status. Therefore, the hemolymph concentration of hexamerin 70a could be used as an additional nutritional biomarker.

Most of these proteins showed an increase from April to November, mirroring the activities of honeybees during the productive season. Differences, though not significant, are evident among the four apiaries, in particular from April to July, suggesting an environmental influence. The study of the effects of the environment around the hives on the nutrition and health status of honeybees is beyond the scope of the present research. However, recent studies have suggested that the landscape can affect bee nutritional physiology and colony performance; in particular, highly managed agricultural landscapes might negatively affect the nutritional physiology of honeybees [9,44]. Accordingly, the data reported in the current study show that the greatest differences between colonies are evident in May, the month in which differences in floral abundance among the environments surrounding the four apiaries are most evident. Bees of apiary C had high concentration of hemolymph proteins coinciding with the peak flowering of spring flora, while in the same month bees of apiary B had very low concentrations of hemolymph proteins, probably due to the shortage of flowering plants in an environment with extensive wheat and sunflower cultivation.

## 3. Materials and Methods

### 3.1. Experimental Design and Hemolymph Sampling

Four apiaries were selected. The apiaries chosen were owned by the same professional beekeeper and were located in different environments of the province of Bologna (Italy): apiaries A (44°32′27.6″ N 11°19′07.4″ E) and D (44°32′37.2″ N 11°17′09.2″ E) were located in the proximity of the town of Bologna, in the proximity of the Reno River, apiary B (44°38′40.6″ N 11°19′49.6″ E) was located on an extensive agricultural landscape characterized by wheat and sunflower, and apiary C (44°22′51.0″ N 11°20′00.3″ E) was in a less anthropized environment, characterized by higher abundance of wild flora. All the colonies did not show any clinical symptom of disease. The colonies were treated for varroosis with Apivar strips (Véto-Pharma, Palaiseau, France) in the absence of the brood. Representative colonies were selected from each apiary based on similar anamnesis (n = 3); all the colonies were homogenous in the number of bees and brood, and honey and pollen stores were adequate. Samplings were performed in the months of April, May, July, and November 2022 to include the entire production season.

For each colony/hive and for each sampling time, after a visual inspection, thirty young worker bees (*Apis mellifera ligustica*) were sampled between the last brood frame and the stores [10] to minimize age variability; in general, nursing bees are found on that frame. The bees were transported to the laboratory of the Department of Veterinary Medical Sciences and 1–2 microliters of transparent uncontaminated hemolymph were collected from each bee as previously described [10]. Briefly, the bees were narcotized with CO_2_ and a glass microcapillary (Blaubrand^®^ BRANDT GMBH, Wertheim, Germany) was inserted between the fourth and fifth tergite to obtain at least one microliter of hemolymph. The hemolymphs collected from honeybees of the same colony were pooled and stored at −80 °C.

### 3.2. Total Protein Determination

Total protein (TP) concentration was measured using the Bradford method (Bradford Reagent, Sigma-Aldrich, MO, USA) following the manufacturer’s instructions. Bovine serum albumin (Sigma-Aldrich, MO, USA) was used as a standard for the calibration curve. The absorbance was measured with a plate reader (Infinite F50 ELISA Plate reader, Tecan Trading AG, Männedorf, Switzerland).

### 3.3. Protein Separation Using SDS-PAGE

Hemolymph proteins were separated using 1D-SDS-PAGE electrophoresis. The diluted hemolymph was loaded onto 4–12% Bis-Tris polyacrylamide gels (NuPage/Thermo Fisher Scientific, Waltham, MA, USA), and electrophoresis was carried out in an Xcell SureLock Mini-Cell with 2-(N-morpholino) propanesulfonic acid buffer (MOPS; NuPage/Thermo Fisher Scientific, Waltham, MA, USA) at pH 7.3 containing sodium dodecyl sulfate (SDS). Different amounts of total proteins from 2 to 15 µg were loaded onto the gel for subsequent identification by mass spectrometry (MS), while to quantify the proteins in the bands of interest, the samples were properly diluted to obtain 3–5 µg of total proteins to be loaded onto the gel. Each gel was also loaded with standard proteins of known molecular mass (SeeBlue™ Plus2 Pre-stained Protein Standard, Thermo Fisher Scientific, Waltham, MA, USA). The electrophoresis system was connected to a power supply (Power Pack Basic—Bio-Rad, Hercules, CA, USA) with a constant voltage of 200 V for 40 min. The gels were stained with Coomassie G250 compatible with MS analysis. After staining, each gel was digitalized using ChemiDocMP (BioRad, Hercules, CA, USA), and pherograms were obtained using ImageLab 5.2.1 software (BioRad, Hercules, CA, USA).

To quantify the protein bands of interest, 1 μg of protein, obtained from a solution containing 1 μg/μL of lactate dehydrogenase (LDH), (Sigma-Aldrich/Merck KGaA, Darmstadt, Germany) was added as an internal standard of quantity to each sample. ImageLab software estimated the volume of each protein band based on pixel density. The volume of the band of interest was then compared to that of the internal standard of the same lane, and the protein content was calculated as previously reported [45].

### 3.4. Protein Identification by Mass Spectrometry

The most represented bands were manually cut from the gel and underwent “in-gel” tryptic digestion, as previously reported [46]. Briefly, the bands were destained with acetonitrile; the proteins were then reduced and subsequently alkylated with dithiothreitol and iodoacetamide, respectively. The proteins were digested with Trypsin (Promega, Madison, WI, USA) at 37 °C, and the peptides obtained were concentrated in a vacuum dryer (Eppendorf Concentrator Plus). The samples were analyzed using a Nano LC-CHIP-MS system (ESI-Q-TOF 6520; Agilent Technologies, Santa Clara, CA, USA) as previously described [10]. Protein identification was obtained using the MASCOT search engine (version 2.7, http://mascot.cigs.unimo.it/mascot (accessed on 20 November 2022)) and the UniProt database, setting the following restriction parameters: taxonomy (a broader taxonomy was selected for identification to be based on sequence homology as the honeybee hemolymph protein database was not well annotated), trypsin as a proteolytic enzyme (max. two missed cleavages), peptide mass tolerance ± 40 ppm and fragment mass tolerance ± 0.12 Da, alkylated cysteine residues (fixed modifications) and oxidized methionine (variable modifications); the significant threshold was set to obtain a false discovery rate of <5%. Only proteins with the highest score hits among the MASCOT search results and identified with at least two or more significant sequences were selected.

### 3.5. Statistical Analysis

Sample distribution was assessed using the Shapiro–Wilk test [47]. The Levene test for homogeneity of variances was applied to each variable at the same time point (month) and among the same groups (apiaries) over the time [48]. Depending on the above-mentioned test outcomes, different statistical tests were applied. One-way ANOVA with Tukey’s HSD post hoc test and the Kruskal–Wallis test one-way ANOVA with Dunn’s multiple comparison post hoc test were performed to determine significant differences among the groups (apiaries) at the same time point [49,50]. The analysis of the variations within the same group at different time points was performed using ANOVA for repeated measures with the pairwise Bonferroni post hoc test or the Friedman rank sum test for repeated measures with the Nemenyi post hoc test [51,52]. In order to apply ANOVA for repeated measures, the presence of outliers was investigated through box-plot visualization [53]. Differences were considered statistically significant for *p* < 0.05. Statistical analyses were performed using R 4.2.2 (R foundation for statistical computing; Vienna, Austria; https://www.R-project.org/, accessed on 1 April 2023). The data are reported as the mean ± SD (standard deviation).

## 4. Conclusions

Overall, in addition to vitellogenin, the application of an expeditious 1D SDS-PAGE protocol to hemolymph proteins allowed the separation and quantification of apolipophorins, transferrin, and hexamerin 70a. These proteins can represent a panel of biomarkers related to key metabolic processes, such as lipid metabolism (apolipophorins) and iron homeostasis (transferrin). Moreover, they are also involved in other important physiological processes, such as nutrition, development, immune response, and longevity (vitellogenin and hexamerins).

In conclusion, this study has suggested a putative panel of hemolymph biomarkers, including total proteins, apolipophorins, vitellogenin, transferrin and hexamerin 70a. These proteins are worth testing in the future under different field conditions to set reference values in healthy colonies, search for a correlation with the strength of the colony (brood, honey, pollen, and parasites), and verify their usefulness in assessing the nutritional and health status of honeybees under stress conditions. The information obtained would be an interesting and innovative management tool to develop beekeeping strategies on a molecular basis.

## Figures and Tables

**Table 1:** Identification of the most abundant proteins in the hemolymph of worker bees from healthy colonies using mass spectrometry.

Band ^1^	Entry Name ^2^	Protein Full Name	MM (Da) ^3^	Score ^4^	Sign. Pept ^5^	Sign. Seq ^6^	emPAI ^7^	Organism
1	A0A088AS56	Apolipophorin (I)	369,557	10,642	579	116	3.07	*Apis cerana*
2	A0A088ADL8	Vitellogenin	202,025	3244	232	54	2.38	*Apis mellifera*
3	A0A088ADL8	Vitellogenin	202,025	34	7	3	0.05	*Apis mellifera*
4	A0A088AS56	Apolipophorin (fragment) (II)	369,557	3848	224	35	2.59	*Apis cerana*
	A0A088AQB0	Leucin-rich repeat-containing protein	76,627	1001	59	13	0.96	*Apis cerana*
5	A0A088AFH7	Transferrin	80,005	1790	103	25	2.92	
6	A5YVK7	Hexamerin 70a	81,522	1429	101	19	2.14	*Apis mellifera*
7	Q86MV4	Prophenoloxidase	80,443	2644	149	20	2.45	*Apis mellifera*
	A0A088AS36	Uncharacterized protein	49,054	94	7	3	0.22	*Apis mellifera*
8	A0A088AMK2	Chitinase-like protein Idgf4	50,442	2708	149	16	4.19	*Apis cerana*
	MRJP1_APIME	Major royal jelly protein 1	49,311	511	16	14	1.48	*Apis mellifera*
9	B0LUE8	Apoliphorin-III like protein	21,335	1723	44	5	3.32	*Apis mellifera*
10	Q1W640	OBP14	15,216	1000	54	6	8.19	*Apis mellifera*
11	Q1W641	OBP13	15,494	77	10	5	1.69	*Apis mellifera*
	Q1W633	OBP21	15,536	112	4	2	0.48	*Apis mellifera*

^1^ The number of the band identified as marked in Figure 1. ^2^ Protein entry name from the UniProt knowledge database. ^3^ Theoretical protein molecular mass. ^4^ The highest scores obtained with the Mascot search engine. ^5^ Significant peptides: total number of significant peptides matching the proteins identified. ^6^ Significant sequences: total number of significant distinct sequences matching the proteins identified. ^7^ Exponentially modified protein abundance index.

## Data Availability

All data analyzed in this study are available upon request.

## Supplementary Materials

The supporting information can be downloaded at: https://www.mdpi.com/article/10.3390/ijms241210216/s1.
