# Supplementary material for: SDS-PAGE-Based Quantitative Assay of Hemolymph Proteins in Honeybees: Progress and Prospects for Field Application
Source: Int J Mol Sci. 2023 Jun 16;24(12):10216. doi: 10.3390/ijms241210216 (PMC10299212; doi:10.3390/ijms241210216)
Supplement: Supplementary file 1 [file ijms-24-10216-s001.zip › ijms-2443949-supplementary/suplementary table S1_EF_GI.pdf]

Supplementary Table S1. Total proteins (TP), apolipophorin-I (ApoLp-I), vitellogenin (Vg), apolipophorin-II (ApoLp-II), transferrin (Tf) and hexamerin 70a (Hex-70a) determined in haemolymph of honeybees sampled from colonies of four apiaries located in the province of Bologna (A, B, C, and D) in different months (April, May, July, a November). Data are expressed in mg/mL and are reported as mean ± SD (n = 3).

		April	May	July	November
TP mg/mL	A	12.7±6.23a	23.3±4.49b,c,d *	19.6±1.70a,c	53.8±12.2b,d
	B	12.6±3.94a	13.3±1.53a,b *	20.4±5.95a,b	49.7±8.53b
	C	13.5±0.36a	44.5±7.50a,b†	21.7±3.58a,b	63.1±3.49b
	D	13.6±3.75a	24.6±9.36a,b *	21.6±9.46a	51.1±10.5b
ApoLp-I mg/mL	A	2.18±0.51	2.03±3.51	7.16±8.36	8.18±1.97
	B	2.37±2.55a,b	0.00±0.00a	3.30±3.26a,b	11.4±0.57b
	C	0.67±0.82	10.0±0.75	5.23±2.50	8.24±1.60
	D	6.56±5.82	2.49±2.98	3.12±7.14	8.73±7.01
Vg mg/mL	A	0.69±0.60a	3.44±5.95a,b	11.9±11.9a,b	33.9±7.13b
	B	0.54±0.66a	0.00±0.00a,b	5.70±5.59a,b	41.5±10.9b
	C	0.39±0.67a	17.5±1.25a	12.2±6.75a,b	38.8±6.95b
	D	3.45±3.51	1.05±0.36	9.33±16.2	35.4±26.6
ApoLp-II mg/mL	A	1.97±0.45	0.29±0.50	2.40±0.62	1.94±0.54
	B	0.82±0.49a,b	0.25±0.03a,b	0.81±0.36a	2.06±0.18b
	C	0.79±0.39	1.68±0.17	2.02±0.83	1.79±0.21
	D	2.39±0.82	1.05±0.36	1.41±2.44	1.21±0.74
Tf mg/mL	A	0.39±0.25a,b	0.29±0.50a	1.24±0.37a,b	2.58±0.51b
	B	0.18±0.08a,b,c	0.07±0.01a	0.56±0.13b	2.32±0.56c
	C	0.21±0.1	2.50±1.29	0.99±0.31	3.18±0.54
	D	0.57±0.49	1.10±0.27	0.74±1.29	2.25±0.72
Hex-70a mg/mL	A	1.55±1.25	0.56±0.97	2.63±1.36	5.36±1.31
	B	0.71±0.33a	0.24±0.02a	1.21±0.74a	4.46±1.14b
	C	0.54±0.27	4.40±0.14	2.37±1.14	6.11±0.40
	D	1.42±1.32	1.70±1.13	1.41±2.44	4.72±0.49

For each analyte, different lower-case letters within rows indicates significant differences ($p<0.05$) among time-points within each apiary.

For each analyte, the different symbols (*; †) within columns indicates significant differences ($p<0.05$) among apiaries within each time-point.
