# Supplementary material for: SDS-PAGE-Based Quantitative Assay of Hemolymph Proteins in Honeybees: Progress and Prospects for Field Application
Source: Int J Mol Sci. 2023 Jun 16;24(12):10216. doi: 10.3390/ijms241210216 (PMC10299212; doi:10.3390/ijms241210216)
Supplement: Supplementary file 1 [file ijms-24-10216-s001.zip › ijms-2443949-supplementary/supplemenary figures-EF_GI.pptx]

## Slide 1
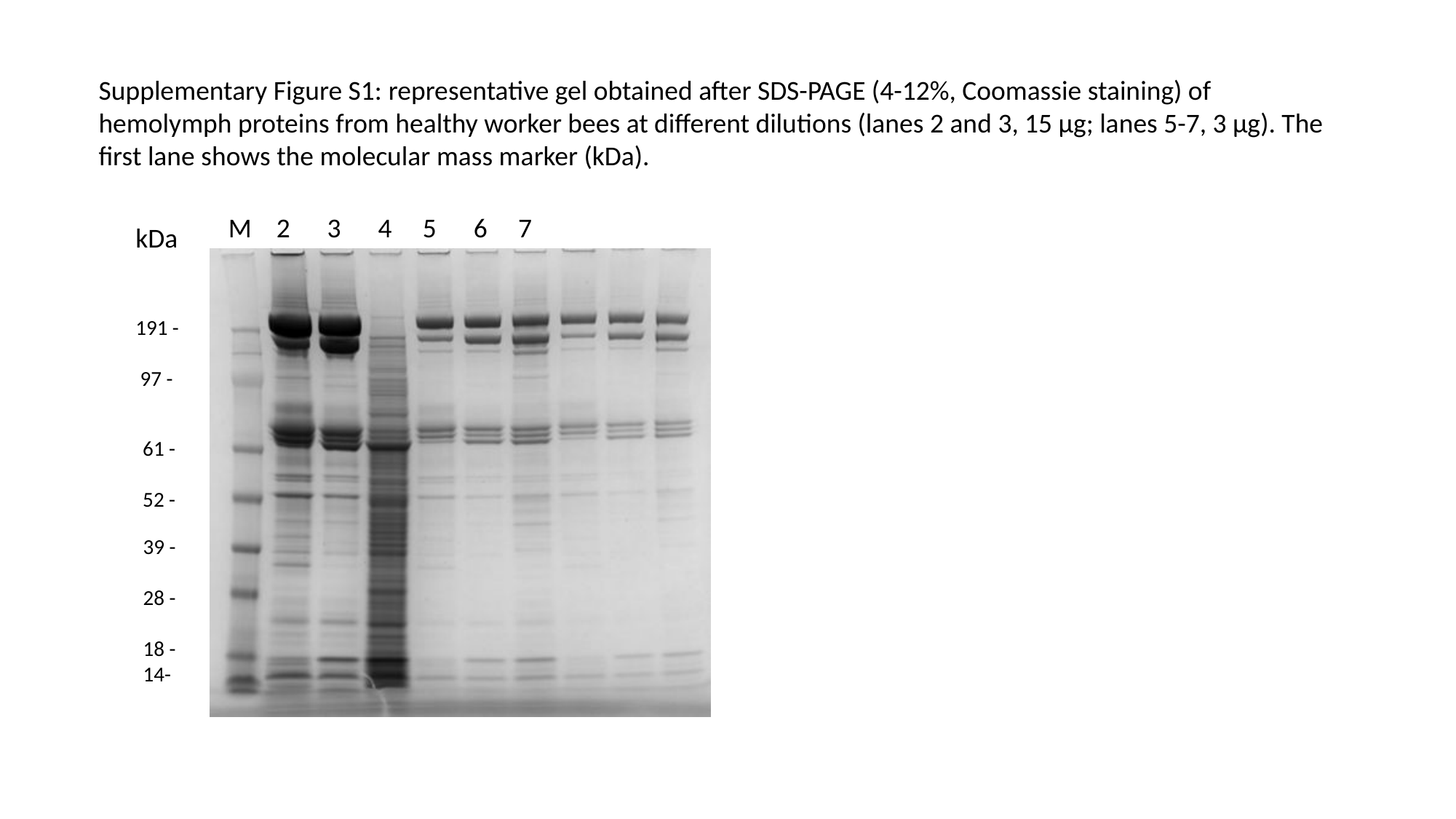

Supplementary Figure S1: representative gel obtained after SDS-PAGE (4-12%, Coomassie staining) of hemolymph proteins from healthy worker bees at different dilutions (lanes 2 and 3, 15 µg; lanes 5-7, 3 µg). The first lane shows the molecular mass marker (kDa).
M 2 3 4 5 6 7
191 -
 97 -
 61 -
 52 -
 39 -
 28 -
 18 -
 14-
kDa

## Slide 2
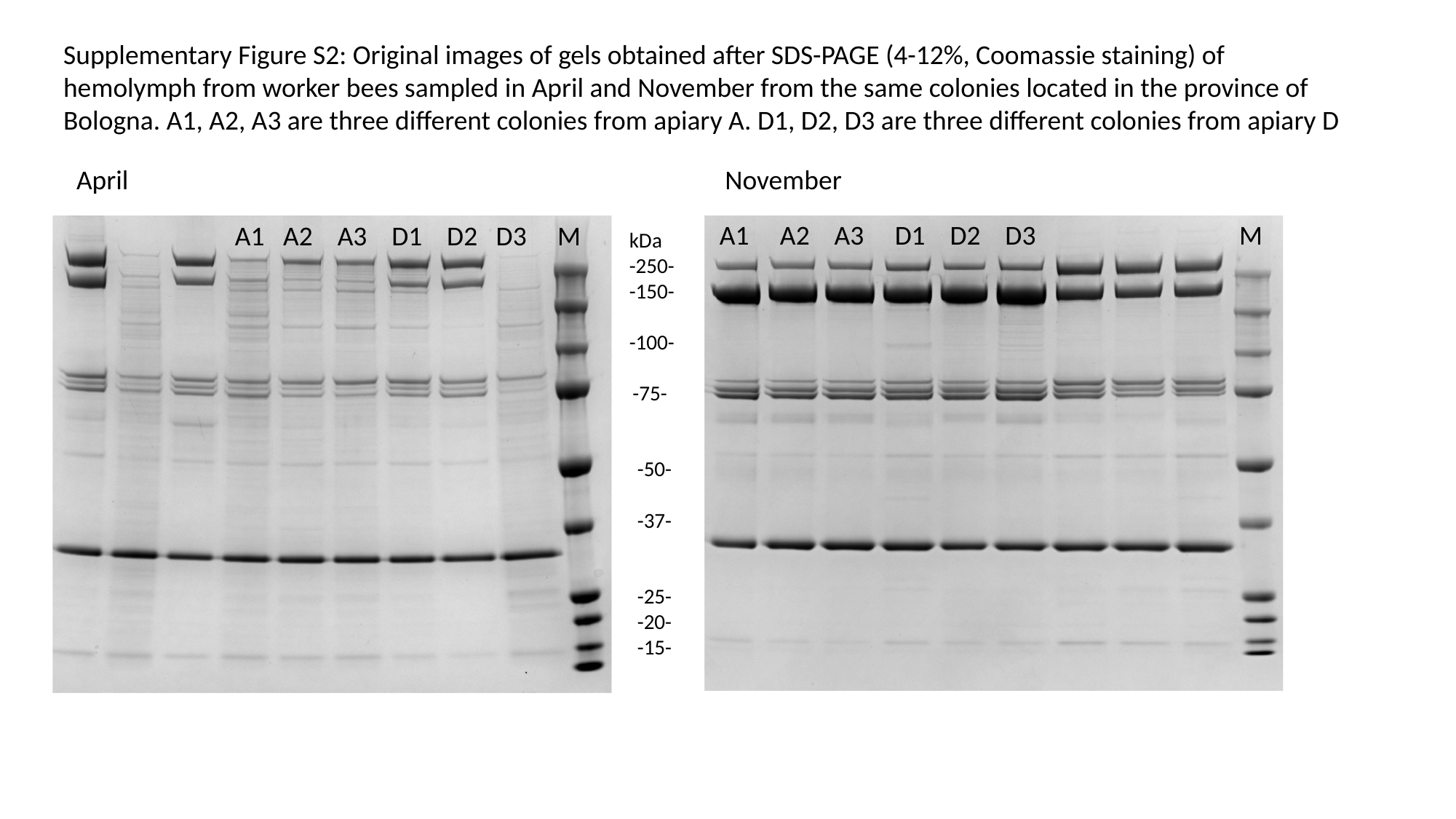

Supplementary Figure S2: Original images of gels obtained after SDS-PAGE (4-12%, Coomassie staining) of hemolymph from worker bees sampled in April and November from the same colonies located in the province of Bologna. A1, A2, A3 are three different colonies from apiary A. D1, D2, D3 are three different colonies from apiary D
April
November
 A1 A2 A3 D1 D2 D3 M
kDa
-250-
-150-
-100-
-75-
 -50-
 -37-
 -25-
 -20-
 -15-
 A1 A2 A3 D1 D2 D3 M
